# Casimir forces exerted by epsilon-near-zero hyperbolic materials

**DOI:** 10.1038/s41598-020-73995-0

**Published:** 2020-10-08

**Authors:** Igor S. Nefedov, J. Miguel Rubi

**Affiliations:** 1 Saratov State University, Astrakhanskaya 83, Saratov, Russian Federation 410012; 2 Departament de Fisica de la Matèria Condensada, Universitat de Barcelona, Marti i Franquès 1, 08028 Barcelona, Spain

**Keywords:** Optics and photonics, Physics

## Abstract

The Casimir force exerted on a gold dipolar nanoparticle by a finite-thickness slab of the natural hyperbolic material namely, the ortorhombic crystalline modification of boron nitride, is investigated. The main contribution to the force originates from the TM-polarized waves, for frequencies at which the parallel and perpendicular components of the dielectric tensor reach minimal values. These frequencies differ from those corresponding to the Lorentzian resonances for the permittivity components. We show that when the slab is made of an isotropic epsilon-near-zero absorbing material the force on the nanoparticle is larger than that induced by a hyperbolic material, for similar values of the characteristic parameters. This fact makes these materials optimal in the use of Casimir’s forces for nanotechnology applications.

## Introduction

The presence of electromagnetic fluctuations is the origin of important phenomena such as thermal emission, radiative heat transfer, van der Waals interactions, Casimir effect and van der Waals (contact-free) friction between bodies^1^ which play an important role in the behavior of matter at very short distances with important implications in nanoscience and nanotechnology. That is why the study of such fluctuations is currently the subject of numerous investigations.

One of the most intriguing effects confirming the foundations of the quantum field theory was predicted by H. Casimir in 1948^2^ and was referred in^3^ to as “*the driving force from nothing*”. In the same year, H. Casimir and D. Polder proposed a theory for dipole interactions taking into accound retardation effects^4^. Experimental confirmation of the existence of Casimir forces was carried out M.J. Sparnaay in 1958^5^. The theory of the Casimir forces for real materials at finite temperatures was proposed by E.M. Lifshitz^6^ and the general theory of Van-der-Waals forces was developed by Dzyaloshinskii, Lifshith and Pitaevskii^7^. It was subsequently shown that the presence of a liquid in between the two interacting bodies may induce a change in the sign of the force that may become repulsive instead of attractive. The methods used to compute Casimir forces and the value of the force for different geommetries has been reviewed in^8–12^.

Futher progress in the study of the Casimir effect and in the Van-der-Waals forces was due to the discovery of new materials such as metamaterials, and to investigations on how micro- and nanoparticles interact with electromagnetic fields. A theory of the Casimir forces under non-equilibrium conditions, for example when the objects are at different temperatures was also developed. These results have been reviewed in^12^.

In the last decade, a number of papers on Casimir forces in hyperbolic metamaterials (HMMs) on the basis of arrays of metal nanowires were published. Metamaterials have shown to be very useful in near-field radiative transfer^13,14^ and in other areas of fluctuation electrodynamics due to the fact that they exhibit stricking properties such as a high density of modes and an ability to support propagating waves with very large wave-vectors. This effect is due to an increase of the density of evanescent fields of plasmonic modes within the gap.

Casimir forces (attractive and repulsive) can act at large distances^15,16^ due to the fact that HMMs may support propagating rather than evanescent waves at large values of the transverse component of the wave vector. The forces are not only perpendicular but also parallel to the surface of the material^17^. Conservative lateral forces induced by corrugations of the surface are local acting only within one of the corrugation periods and a consequence, they can only exert local lateral displacements. On the contrary, non-conservative lateral forces as the ones induced by fluctuating currents in an anisotropic HMM (boron nitride) can move a particle persistently along a flat homogeneous boundary^20^. The lateral movement of anisotropic particles along a surface was analyzed in^18^. The motion, however, ceases when the particle adopts an orientation for which its energy is a minimum. Lateral Casimir forces can also cause particle rotation^19^.

The interest in metamaterials with near-zero (NZ) parameters (refractive index, permittivity and permeability) is due to the fact that structures made of these materials offer enormous possibilities for applications^21^. Near-zero-index photonics is currently an area of rapid growth^22^. Structures with near-zero parameters are: epsilon-near-zero (ENZ), $\epsilon \approx 0$, mu-near-zero (MNZ), $\mu \approx 0$, and epsilon-mu-near-zero (EMNZ)^22^. All of these cases exhibit a near-zero index of refraction: $n=\sqrt{\epsilon \mu }\approx 0$.

Ziolkovski proposed zero index of refraction metamaterials for the decoupling of spatial and temporal variations of the field^23^. This idea is based on the fact that the field in the region with near-zero parameters exhibits a static-like character even if it oscillates in time. Tunneling of electromagnetic waves through a narrow two-dimensional channel filled with an ENZ material was predicted in^24^. Near-zero-index media can enhance nonlinear processes^25,26^, optical activity in one-dimensional epsilon-near-zero pseudochiral metamaterials^27^, electric levitation^28^ and other field-matter interaction processes.

In this article, we compute the Casimir force on a gold dipolar nanoparticle induced by a slab of an $\epsilon$-near-zero hyperbolic and isotropic material and show that its main contribution comes from the frequency domain where $\epsilon \approx 0$.

The article is organized as follows. In Section II, we introduce the model and analyze the different contributions to the radiative force on a gold particle close to a slab of an absorptive anisotropic material. In Section III, we present our results of the Casimir force and in Section IV we summarize our main conclusions.

## The model

We consider a slab made of an absorptive anisotropic material which is infinite in the *x*- and *y*-directions and has thickness *h* in the *z*-direction. The anisotropy axis is directed along the *z*-axis. We will compute the Casimir forces acting on a gold nanoparticle placed nearby the slab. The relative permittivity tensor of the material has the diagonal form

1 $$\begin{aligned} \overline{\overline{\epsilon }}=\epsilon _{\parallel }\mathbf{z}_0\mathbf{z}_0+\epsilon _t(\mathbf{x}_0\mathbf{x}_0+\mathbf{y}_0\mathbf{y}_0)\end{aligned}$$

where $\mathbf{x}_0,\,\mathbf{y}_0,\,\mathbf{z}_0$ are the coordinate unit vectors.

This particular geometry allows us to analyze separately the propagation of TM and TE waves in the slab. Let us consider the TM modes and find fields excited by point-like fluctuating currents within the slab in the frequency domain. For the tangential field components $\mathrm{X}(z)=\left( E_x(z),H_y(z)\right)$, excited by the fluctuating currents $j_x(z),\,j_z(z)$ located within the absorptive layer $0<z<h$, the Maxwell equations reduce to the system of two ordinary differential equations:

2 $$\begin{aligned} \frac{d}{dz}\mathrm{X}(z)=[\mathrm{A}] \mathrm{X}(z)+\mathrm{F}(z)\end{aligned}$$

with the matrix elements of [A] given by

3 $$\begin{aligned} \begin{array}{lr} A_{11}=0, &{} A_{12}=i\eta k_0\left( k_0\mu -\frac{k_x^2}{k_0^2\epsilon _{\parallel }}\right) \\ A_{21}=i\frac{k_0}{\eta }\epsilon _{\perp }, &{} A_{22}=0, \end{array}\end{aligned}$$

where $k_0$ and $\eta =120\pi$ Ohm are the wavenumber and wave impedance in vacuum, respectively. The components of the vector F(*z*) = $(F_1(z),F_2(z))$ are

4 $$\begin{aligned} \begin{array}{l} F_1(z)=\eta \frac{k_x}{k_0\epsilon _{\parallel }}j_z(z)=aj_z(z), \\ F_2(z)=-j_x(z). \end{array}\end{aligned}$$

The elementary bulk current source has the form: $\mathbf{j}(z)=\mathbf{j}_0(z')\delta (z-z')$.

The solution of Eq. (2) for points in the interval $0<z<h$ is^34^:

5 $$\begin{aligned} \mathrm{X}(z)=e^{[\mathrm{A}]z}\mathrm{X}(0)+\int _0^z{e^{[\mathrm{A}](z-\tau )}\mathrm{F}(\tau )}d\tau ,\end{aligned}$$

with $[\mathrm{M}(z)]=e^{[\mathrm{A}]z}$ the transfer matrix:

6 $$\begin{aligned} \begin{array}{lr} M_{11}(z)=\cos {k_zz} &{} M_{12}(z)=iZ \sin {k_zz}\\ M_{21}(z)=\frac{i}{Z}\sin {k_zz} &{} M_{22}(z)=M_{11}(z)\end{array}\end{aligned}$$

where $k_x$ and $k_z=\sqrt{\epsilon _{\perp }(k_0^2-\frac{k_x^2}{\epsilon _{\parallel }})}$ are the transverse and normal components of the wave vector in the slab, respectively, and

7 $$\begin{aligned} Z=\eta \frac{k_z}{k_0\epsilon _\perp } \end{aligned}$$

is the transverse wave impedance for the TM mode. The boundary conditions are: $X_2(0)=X_1(0)/Z_0,\;X_2(h)=-X_1/Z_0$, where $Z_0=\eta \sqrt{(k_0^2-k_x^2)}/k_0$ is the transverse wave impedance in vacuum. We can then express the tangential field components at the interface $x=0$, created by a current located at $z'$ in the form

8 $$\begin{aligned} \begin{array}{l} X_1(0,z')=\frac{1}{\Delta }\int _0^h\left[ U(\tau )j_{x0}(z')+ V(\tau )j_{z0}(z')\right] \delta (\tau -z')\,d\tau \\ \quad =\frac{1}{\Delta }\left[ U(z')j_{x0}(z')+V(z')j_{z0}(z')\right] , \end{array} \end{aligned}$$

where

9 $$\begin{aligned} \begin{array}{l} \Delta =M_{11}(h)+M_{22}(h)-M_{12}(h)/Z_0-M_{21}(h)Z_0, \\ U(\tau )=iZ\sin {k_z(h-\tau )}-Z_0\cos {k_z(h-\tau )} \\ V(\tau )=\eta \frac{1}{k_0\epsilon _\parallel }\left[ i\frac{Z_0}{Z}\sin {k_z(h-\tau )} -\cos {k_z(h-\tau )} \right] . \end{array} \end{aligned}$$

The average values of the fluctuating currents vanish, only their correlations contribute to the energy flux. These correlations are given through the fluctuation-dissipation theorem^35^. The ensemble-averaged correlator $\langle \mathbf{E}(\omega ,k_x,z')\mathbf{E}^*(\omega ,k_x,z'') \rangle$ in the plane $z=0$, for the $k_{x}$ mode, induced by fluctuating currents located within the slab, $0<z',z''<h$, reads

10 $$\begin{aligned}\langle \mathbf{E}(\omega ,k_x)\mathbf{E}^*(\omega ,k_x)\rangle =\frac{1}{2}\int _0^h\int _0^h\langle \mathbf{E}(\omega ,k_x,z^{\prime})\mathbf{E}^*(\omega ,k_x,z^{\prime\prime})\rangle \,dz^{\prime}dz^{\prime\prime}, \end{aligned}$$

where the correlation $\langle \mathbf{E}(\omega ,k_x,z')\mathbf{E}^*(\omega ,k_x,z'') \rangle$ is obtained through the fluctuation-dissipation theorem^35^

11 $$\begin{aligned} \langle j_m(\mathbf{r},\omega )j^*_n(\mathbf{r}^{\prime},\omega^{\prime})\rangle = \frac{4}{\pi }\omega \epsilon _0\epsilon _{mn}^{\prime\prime}(\omega )\delta (\mathbf{r}-\mathbf{r}^{\prime})\delta (\omega -\omega ^{\prime})\Theta (\omega ,T), \end{aligned}$$

with $$\mathbf{r}=(x,z)$$ and

12 $$\begin{aligned} \Theta (\omega ,T)=\hbar \omega \left( \frac{1}{2}+\frac{1}{e^{\hbar \omega /(k_BT)}-1}\right) \end{aligned}$$

the Planck’s oscillator energy. In Eq. (11), $\epsilon _{mn}''\equiv \mathrm{Im}(\epsilon _{mn})$, $\epsilon _0$ is the permittivity of vacuum, $\hbar$ the reduced Planck constant, *T* the temperature, and $k_B$ the Boltzmann constant.

We will consider radiative forces on a small nanoparticle in the dipole approximation. The dipolar force acting on the particle can be written as

13 $$\begin{aligned}&\langle \mathbf{F}\rangle =\frac{1}{4}\mathrm{Re}\{\alpha \}\nabla |\mathbf{E}|^2+ \sigma \frac{1}{2}\mathrm{Re}\left\{ \frac{1}{c} \mathbf{E}\times \mathbf{H}^*\right\} \\&\quad +\sigma \frac{1}{2}\mathrm{Re}\left\{ i\frac{\epsilon _0}{k_0}(\mathbf{E}\cdot \nabla )\mathbf{E}^*\right\} \end{aligned}$$

This formula was derived from the Maxwell stress tensor for the dipolar particle^36^. In it, $\alpha$ is the polarizability of the particle given by

14 $$\begin{aligned} \begin{array}{ll} \alpha =\frac{\alpha _0}{1-i\alpha _0k_0^3/(6\pi \epsilon _0)},&\alpha _0=4\pi \epsilon _0r^3\frac{\epsilon -1}{\epsilon +2}, \end{array}\end{aligned}$$

with *r* and $\epsilon$ its radius and permittivity, respectively, and $\sigma =k_0\mathrm{Im}\{\alpha \}/\epsilon _0$.

The first term in (13), related to the gradient forces, causes attraction of the particle toward the slab interface due to the *z*-dependence of the fields through the factor $e^{|k_{z0}|z}$, for $z<0$. This term properly defines the Casimir or the van-der-Waals forces on a nanoparticle^1^. The explicit expression for $\nabla |\mathbf{E}|^2$ is

15 $$\begin{aligned} \begin{array}{l} \nabla |\mathbf{E}(\omega ,k_x,z)|^2=\frac{\partial }{\partial z}f(z)\left[ \langle E_xE_x^*\rangle +\langle E_zE_z^*\rangle \right] \end{array}\end{aligned}$$

where $$f(z)=1$$, if $$|k_x|<k_0$$, and $$f(z)=e^{2|k_{z0}|z}$$$$(z<0)$$, if $$|k_x|>k_0$$^20^. Only evanescent waves $$(|k_x|>k_0)$$ contribute to this force.

In the second contribution, the *x*- and *z*-component of the Poynting vector corresponds to pulling forces along the corresponding directions. The *x*-component of the Poynting vector integrated over $k_x$ is zero in the case of a symmetric geometry and different from zero in the asymmetric case, as shown in^20^. At small |*z*|, the attractive gradient force is dominant whereas at larger |*z*| the dominant force is the repulsive force proportional to the *z* component of the Poynting vector. Very often, this contribution is considered as a part of the Casimir force. The third term in (13) does not contribute to radiative forces^20^.

Using the fluctuation-dissipation theorem (11), and expression (15) and integrating over *z*, as done in^20^ [see formulas (17)], we obtain

16 $$\begin{aligned} \langle F_z(\omega ,k_x)\rangle = \frac{4\omega \epsilon _0\Theta (\omega ,T)}{2\pi |\Delta |^2}\left[ D_1\epsilon _{xx}''+D_2\epsilon _{zz}''\right] ,\end{aligned}$$

where we have defined the coefficients

17 $$\begin{aligned} \begin{array}{l} D_1=|Z|^2S+|Z_0|^2C+2\mathrm{Im}(ZZ_0^*G) \\ D_2=|a|^2\left[ \left| \frac{Z_0}{Z}\right| ^2S+C+2\mathrm{Im}\left( \frac{Z_0}{Z}G\right) \right] \end{array}\end{aligned}$$

with

18 $$\begin{aligned} \begin{array}{l} C=\int _0^h{|\cos {[k_z(h-\tau )]}|^2}d\,\tau \\ S=\int _0^h{|\sin {[k_z(h-\tau )]}|^2}d\,\tau \\ G=\int _0^h{\cos {[k_z^*(h-\tau )]}\sin {[k_z(h-\tau )]}}d\,\tau . \end{array}\end{aligned}$$

The non-conservative force, determined by the second term of (13), proportional to the Poynting vector, is negligible at small distances from the boundary of an absorptive medium.

The Casimir force exerted by the TM waves is obtained by integrating the force over $k_x$ and $\omega$:

19 $$\begin{aligned} \langle F_z\rangle =\frac{1}{4\pi ^2}\int _{-\infty }^{\infty } \int _{-\infty }^{\infty }{\langle F_z(\omega ,k_x)\rangle } k_x\,dk_xd\omega . \end{aligned}$$

## Results and discussion

Cubic, hexagonal, rhombohedral and orthorhombic cristalline forms of boron nitride exhibit hyperbolic dispersion in the infrared frequency range^29–32^. As a particular case, we will consider the orthorhombic form. The components of the permittivity tensor are given by the Lorentz model^32^:

20 $$\begin{aligned} \epsilon _{\parallel ,\perp }=\epsilon _{\parallel ,\perp }^{\infty }+ \frac{U_{\parallel ,\perp }(\omega _{\parallel ,\perp }^{\tau })^2}{(\omega _{\parallel ,\perp }^{\tau })^2-\omega ^2-i\omega \Gamma _{\parallel ,\perp }}, \end{aligned}$$

where $\omega _{\parallel ,\perp }^{\tau }$ and $U_{\parallel ,\perp }$ are, respectively, the transverse phonon frequency and the oscillator strength of the lattice vibration for the parallel and perpendicular polarizations, and $\Gamma _{\parallel ,\perp }$ is the damping constant. The constants $\epsilon _{\parallel ,\perp }^{\infty }$ are the components of the permittivity tensor at frequencies $\omega$ that greatly exceed the phonon resonance frequency $\omega _{\parallel ,\perp }^{\tau }$. The values of the parameters of (20) used are: $\epsilon _{\parallel }^{\infty }=2.7$, $U_{\parallel }=0.48$, $\omega _{\parallel }^{\tau }=1.435\times 10^{14}$ rad/s, $\Gamma _{\parallel }=8.175\times 10^{11}$ rad/s, $\epsilon _{\perp }^{\infty }=5.2$, $U_{\perp }=2$, $\omega _{\perp }^{\tau }=2.588\times 10^{14}$ rad/s, $\Gamma _{\perp }=1.29\times 10^{12}$ rad/s. For these values of the parameters, the Lorentzian resonances of $\epsilon _{\parallel }$ and $\epsilon _{\perp }$ take place at frequencies $\approx 22.8$ THz and $\approx 41.2$ THz, respectively. In the vicinity of these resonances, the real parts of $\epsilon _{\parallel }$ and $\epsilon _{\perp }$ change their signs and the imaginary parts are very large. One can expect an increase of the Casimir forces near the $\epsilon _{\perp }$ resonance due to the singularity of $1/|\Delta |^2$ if $|\epsilon _{\perp }|^2\rightarrow 0$. Similarly, an increase of the Casimir force per unit of frequency is expected near the $\epsilon _{\parallel }$ resonance when $|V(\tau )|^2$ increases due to the increase of $|a|^2=|\eta k_x/(k_0\epsilon _{\parallel })|^2$, if $\epsilon _{\parallel }\rightarrow 0$ [see Eq. (9)].

**Figure 1:**
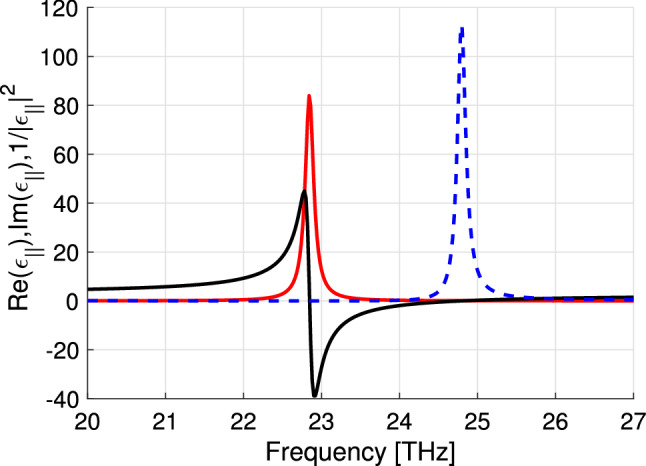
Re($\epsilon _\parallel$) (black solid line), Im($\epsilon _\parallel$) (red solid line), and $1/|\epsilon _\parallel |^2$ (blue dashed line).

**Figure 2:**
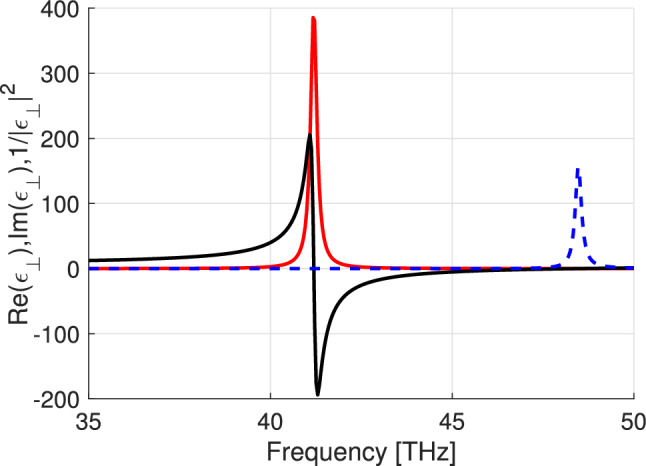
Re($\epsilon _\perp$) (black solid line), Im($\epsilon _\perp$) (red solid line), and $1/|\epsilon _\perp |^2$ (blue dashed line).

Figure 1 illustrates the frequency dependence of Re($\epsilon _\parallel$) and Im($\epsilon _\parallel$) on the frequency range where the corresponding permittivity component experiences the Lorentzian resonance. Because of the losses, $|\epsilon _{\parallel }|^2\rightarrow 0$ near the frequency 24.9 THz. Figure 2 shows similar dependencies for the perpendicular component of the permittivity. Here, we see that $|\epsilon _{\perp }|^2\rightarrow 0$ near 48.5 THz.

As an example of particle, we consider a spherical gold nanoparticle whose complex permittivity $\epsilon _g$ in the infrared range is, according to the Drude model, given by

21 $$\begin{aligned} \epsilon _g=\epsilon _{\infty }-\frac{\omega _p^2}{\omega ^2+i\omega \omega _r}, \end{aligned}$$

where $\omega _p=1.367\times 10^{16}$ rad/s and $\omega _r=10.5\times 10^{13}$ rad/s are the plasma frequency and the damping frequency, respectively and $\epsilon _{\infty }=9.5$^33^. The radius of the particle is 10 nm.

**Figure 3:**
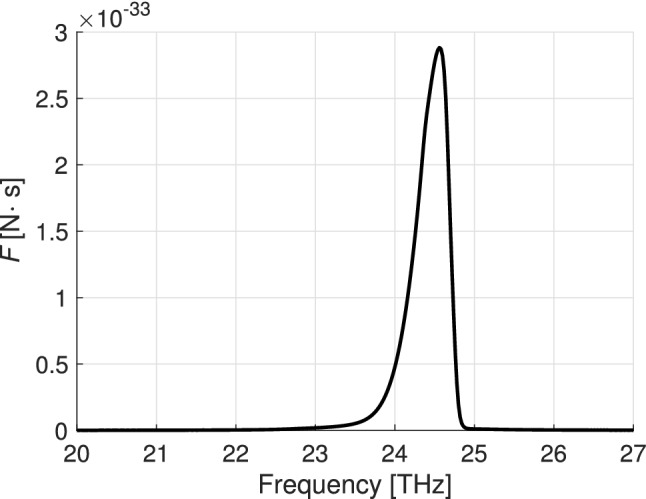
The Casimir force per frequency unit versus frequency in the vicinity of the $\epsilon _\parallel$-near-zero region. $h=300$ nm, $T=450$ K, $z=100$ nm.

**Figure 4:**
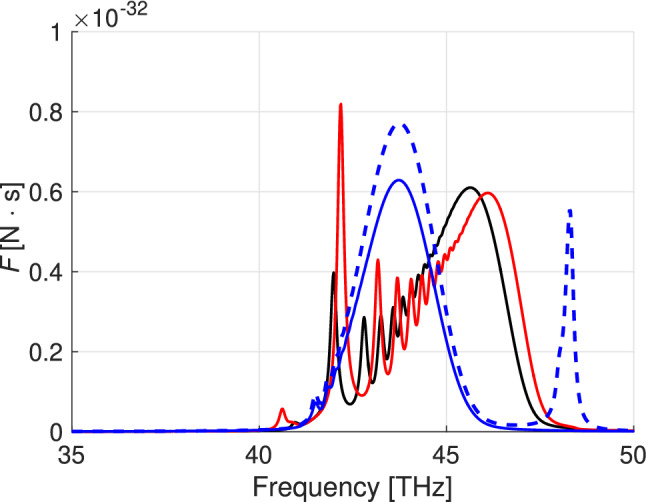
Casimir force per frequency unit versus frequency in the vicinity of the $\epsilon _{\perp }$-near-zero region calculated for the thickness $h=400$ nm (red), $h=300$ nm (black), and $h=100$ nm (blue). Dashed blue line shows the Casimir force per frequency unit calculated for an isotropic material with permittivity $\epsilon =\epsilon _{\perp }$, at $h=100$ nm.

As expected, the main contribution to the Casimir forces comes from the regions where $|\epsilon _{\parallel }|\rightarrow 0$ and $|\epsilon _{\perp }|\rightarrow 0$. Figs. 3 and 4 show the Casimir force per frequency unit in the vicinity of these frequencies. Oscillations in Fig. 4 are caused by the excitation of plasmon-polaritons in the vicinity of $\epsilon$-near-zero frequencies^38,39^. For hyperbolic materials $|k_{z}|$ becomes very large at frequencies $\omega <\omega _0$, where $\epsilon (\omega _0)\approx 0$. At these frequencies in a finite-thickness slab exists a dense (countable in the lossless limit) spectrum of modes (see^40^, Fig. 14) which manifest itself as the ‘fringes’ in Fig. 4.

**Figure 5:**
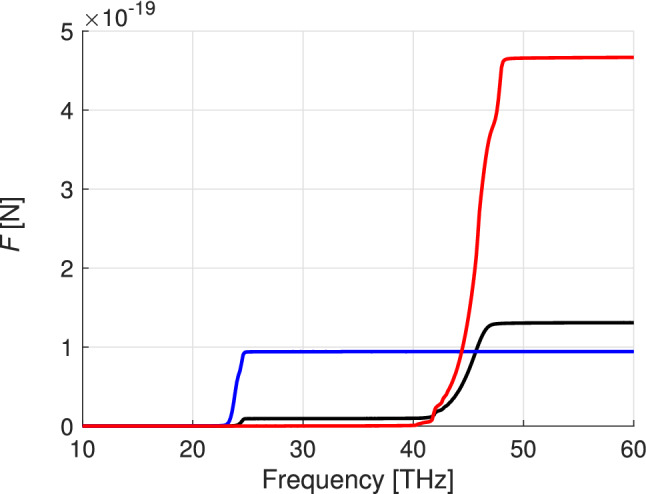
The Casimir force [N] versus frequency calculated for boron nitride (black), for a material with permittivity $\epsilon =\epsilon _{\perp }$ (red), and for a material with $\epsilon =\epsilon _{\parallel }$ (blue).

Figure 5 shows the overall Casimir force. The lower integration limit over frequency is 10 THz since at smaller frequencies contributions to the Casimir force are very small. The upper limit corresponds to a frequency at the abscisa axis. The result of the integration increases dramatically in the frequency domains where $|\epsilon _{\parallel }|$ and $|\epsilon _{\perp }|$ are minimal. The repulsive force due to the second term in (13) (proportional to the Poynting vector) is of order $10^{-23}$ N. In the figure , we compare the Casimir forces exerted by the slab of hyperbolic material (boron nitride) to the ones obtained from a hypothetical isotropic material with permittivities $\epsilon =\epsilon _{\parallel }$ and $\epsilon =\epsilon _{\perp }$. The greatest Casimir force is induced by the isotropic material with the permittivity undergoing the Lorentzian resonance for $\epsilon _{\perp }$ in boron nitride.

## Conclusions

In summary, we have shown that the main contributions to the Casimir forces on a dipolar particle results from the TM-polarized waves and takes place at regions where $|\epsilon _{\parallel }|$ and $|\epsilon _{\perp }|$ are minimal which differ from the regions at which the Lorentzian resonances for the corresponding permittivity components take place. The leading contribution comes from the $|\epsilon _{\perp }|$-near-zero region. Hyperbolicity itself (i.e. different signs of the parallel and the perpendicular components of the permittivity) does not guarantee a high force value, compared to the one obtained for an $\epsilon$-near-zero isotropic absorbing material corresponding to the TE-waves and excluding the term with $\epsilon _{\parallel }''$.

Our result of the Casimir force differs from that obtained in the case that both objects are made of the same HMM^37^ in which the force is much greater than that obtained between dielectric materials. This fact indicates that the nature of the materials and the frequency dependence of the permittivity could play a role in the value of the force. The effect of TE-waves in the Casimir force was also analyzed arriving at the conclusion that the force is three orders of magnitude smaller than the one resulting from the TM-waves.

The enhancement of the Casimir force found when ENZ hyperbolic materials are used shows that these materials could be advantageous for the use of Casimir’s forces in nanotechnology.
