# Author Correction: Casimir forces exerted by epsilon-near-zero hyperbolic materials

**DOI:** 10.1038/s41598-020-78860-8

**Published:** 2020-12-09

**Authors:** Igor S. Nefedov, J. Miguel Rubi

**Affiliations:** 1 Peoples’ Friendship, University of Russia (RUDN University), 6 Miklukho-Maklaya St., Moscow, Russia 117198; 2 Departament de Fisica de la Matèria Condensada, Universitat de Barcelona, Marti i Franquès 1, 08028 Barcelona, Spain

Correction to: *Scientific Reports* 10.1038/s41598-020-73995-0, published online 08 October 2020

In the original version of this Article, Igor S. Nefedov was incorrectly affiliated with “Saratov State University, Astrakhanskaya 83, Saratov, Russian Federation, 410012”. The correct affiliation is listed below:

Peoples’ Friendship University of Russia (RUDN University), 6 Miklukho-Maklaya St., Moscow 117198, Russia.

This error has now been corrected in the HTML and PDF versions of the Article.

